# Calsarcin-2 May Play a Compensatory Role in the Development of Obese Sarcopenia

**DOI:** 10.3390/biomedicines11102708

**Published:** 2023-10-05

**Authors:** Yu-Cheng Liang, Kai-Pi Cheng, Hsin-Yu Kuo, Chung-Teng Wang, Hsuan-Wen Chou, Kuan-Lin Huang, Hung-Tsung Wu, Horng-Yih Ou

**Affiliations:** 1 Department of Internal Medicine, National Cheng Kung University Hospital, College of Medicine, National Cheng Kung University, Tainan 704, Taiwan; ycliang.official@gmail.com (Y.-C.L.); supercabyhome@gmail.com (K.-P.C.); telomere-aging@hotmail.com.tw (H.-Y.K.); coolpikachu2007@gmail.com (H.-W.C.); 2 Department of Internal Medicine, School of Medicine, College of Medicine, National Cheng Kung University, Tainan 701, Taiwan; knight790105@hotmail.com; 3 Department of Biochemistry and Molecular Biology, College of Medicine, National Cheng Kung University, Tainan 701, Taiwan; ken.huang1@astrazeneca.com

**Keywords:** calsarcin-2, high-fat diet, myoblast differentiation, myocyte, sarcopenia

## Abstract

Although obese sarcopenia is a major public health problem with increasing prevalence worldwide, the factors that contribute to the development of obese sarcopenia are still obscure. In order to clarify this issue, a high-fat-diet-induced obese sarcopenia mouse model was utilized. After being fed with a high-fat diet for 24 weeks, decreased motor functions and muscle mass ratios were found in the C57BL/6 mice. In addition, the expression of calsarcin-2 was significantly increased in their skeletal muscle, which was determined by a microarray analysis. In order to clarify the role of calsarcin-2 in muscle, lentiviral vectors containing the calsarcin-2 gene or short hairpin RNA targeted to calsarcin-2 were used to manipulate calsarcin-2 expressions in L6 myoblasts. We found that an overexpression of calsarcin-2 facilitated L6 myoblast differentiation, whereas a calsarcin-2 knockdown delayed myoblast differentiation, as determined by the expression of myogenin. However, the calsarcin-2 knockdown showed no significant effects on myoblast proliferation. In addition, to clarify the relationship between serum calsarcin-2 and sarcopenia, the bilateral gastrocnemius muscle mass per body weight in mice and appendicular skeletal muscle mass index in humans were measured. Although calsarcin-2 facilitated myoblast differentiation, the serum calsarcin-2 concentration was negatively related to skeletal muscle mass index in mice and human subjects. Taken together, calsarcin-2 might facilitate myoblast differentiation and appear to play a compensatory role in sarcopenia.

## 1. Introduction

Sarcopenia is a multifactorial disease that is defined by both muscle mass loss and strength due to aging, eating habits, physical inactivity, or other diseases [1,2,3,4]. The prevalence of sarcopenia among people over 55 years old is 9.9–40.4%, depending on the population surveyed and the diagnostic criteria used [5]. The disease has been associated with a poorer quality of life [6], functional decline, higher rates of falls and hospitalization, and higher all-cause mortality [7,8].

The physiological function of muscle is not only important for motor activity but also plays an important role in energy metabolism and glucose uptake [9]. Dysfunction of skeletal muscle, such as sarcopenia, is highly associated with chronic metabolic diseases such as diabetes, metabolic syndrome, and obesity [10]. Thus, the coexistence of sarcopenia and obesity has recently emerged as a new public health problem known as sarcopenic obesity [11]. It is estimated that sarcopenic obesity will affect 100–200 million people in the next 35 years [12], and the medical sequelae related to sarcopenic obesity are much greater than simple sarcopenia or obesity alone, resulting in considerably higher healthcare costs [13]. Although resistance-based exercises and a quality protein-rich diet/protein supplementation, as well as Vitamin D supplementation, are recommended as a therapeutic strategy for the treatment of sarcopenia, the effectiveness of these treatments still needs further evaluation [14]. In addition, the factors that contribute to the development of sarcopenia remain obscure.

Several aspects are involved in the pathophysiology of sarcopenia. A recent review showed there is a trend of muscle fiber type-II-to-I shifting in sarcopenia during the aging process [15]. It has been known that the muscle-specific RING finger (MuRF) family plays a critical role in skeletal muscle degradation and maintenance [16]. A previous study revealed that MuRF1 and MuRF2 together are required to maintain type-II muscle fiber, probably through the regulation of calsarcin-2 [17].

Calsarcin is a 32 kDa muscular sarcomere microstructure protein, and its transcript is expressed primarily in skeletal muscle, with significantly lower levels of expression in several other tissues [18,19,20]. The Calsarcin gene has six exons and maps to the human chromosome 10q22.1-q22.2, and it inhibits calcineurin signaling, thereby reducing the type-I muscle fiber content in skeletal muscle [21,22]. Additionally, it has been postulated that calsarcin-2 may be involved in regulating the cell fate of myoblasts [23]. Thus, calsarcin is a skeletal muscle Z line protein that may be a good candidate gene for limb-girdle muscular dystrophy or other neuromuscular disorders. However, the role of calsarcin-2 in the development of sarcopenia is still obscure. Furthermore, a prior study unveiled an elevation in calsarcin-2 expression among men with metabolic syndrome [24]. Given that metabolic syndrome stands as one of the key indicators in screening for sarcopenic obesity [25], the correlation between sarcopenic obesity and calsarcin-2 warrants thorough examination.

In this study, we used a high-fat diet (HFD)-induced obese sarcopenia animal model and measured the expression of calsarcin-2 in muscle and serum calsarcin-2 levels. In addition, we used the L6 myoblast cell line to investigate the cellular functions of calsarcin-2 and clarify the possible role of calsarcin-2 in sarcopenia.

## 2. Materials and Methods

### 2.1. Animals

All animal procedures (IACUC No:108213) were performed according to the guidelines of the Animal Welfare Act as well as the Guide for the Care and Use of Laboratory Animals from the National Institutes of Health. Eight-week-old C57BL/6J male mice were purchased from the Animal Center at the National Cheng Kung University Medical Center and housed in a temperature- (25 ± 1 °C) and humidity- (60 ± 5%) controlled room with a 12:12 light–dark cycle (lights on at 07:00 AM), and the animals were randomly divided into chow and HFD groups (*n* = 6–8 for each group of the mice). The mouse model of obese sarcopenia was established by feeding them an HFD containing 34.9% fat (58Y1, TestDiet, St. Louis, MO, USA) for 24 weeks, according to previous studies [26,27]. The blood samples were collected for the determination of calsarcin-2 levels (MyBiosource, San Diego, CA, USA) using commercial assay kits after the mice fasted for twelve hours. At the end of the experiments, each group of mice was euthanized, and gastrocnemii were collected. The sum of the bilateral gastrocnemius muscle mass divided by the weight of the mouse was used as an index of its skeletal muscle mass.

#### 2.1.1. Grip Strength Test

The mice were gently held at the tail base over the top of the grid and allowed to grip the grid with their forelimbs and hindlimbs. The mice were then pulled back with their torsos in a horizontal position. The maximum force (at the time the mice released their grasp) was recorded using a grip strength meter. The values for grip strength were obtained from five consecutive trials with one-minute inter-trial intervals.

#### 2.1.2. Rotarod Test

The mice kept their balance on a rotating rod (3 cm diameter), with speed accelerating from 4 to 40 rpm over the course of 300 s (Ugo Basile, Como, Italy). The time taken to fall from the rotating rod was recorded. Three consecutive trials were performed with one-minute inter-trial intervals, and the mean time to fall was calculated.

#### 2.1.3. Determination of Skeletal Muscle Cross-Sectional Area

The mid-belly region of the gastrocnemius muscles from each group of mice were collected and fixed in phosphate-buffered saline (PBS) containing 10% formaldehyde. Fixed specimens were dehydrated and embedded in paraffin. The samples were then cut into sections of 5 μm thickness at 5 μm intervals and stained with hematoxylin and eosin (H&E). The cross-sectional area of each muscle fiber was measured using an automated function in ImageJ software from W. Rasband (http://rsb.info.nih.gov/ij/; accessed on 21 May 2022; National Institutes of Health, Bethesda, MD, USA) [28].

### 2.2. Western Blot Analyses

Samples were lysed in ice-cold radioimmunoprecipitation assay buffer with a protease inhibitor cocktail and a phosphatase inhibitor cocktail (Sigma-Aldrich, St. Louis, MO, USA). A bicinchoninic acid assay kit (Pierce Biotechnology, Waltham, MA, USA) was used to determine the concentrations of proteins. Sodium dodecyl sulfate-polyacrylamide gel electrophoresis was performed to separate the protein samples (30 μg), and the samples were then transferred to a polyvinylidene difluoride membrane (Millipore, Billerica, MA, USA). Subsequently, the membrane was blocked at room temperature for one hour in Tris-buffered saline with Tween 20 (TBS-T), containing 10% skim milk, and probed with primary antibodies (1:1000 dilutions), including calsarcin-2, myogenin (MYOG), myoblast determination protein 1 (MYOD) (Abcam, Cambridge, UK), and pan-actin (Millipore) at 4 °C overnight. The blots were then washed with TBS-T and incubated with horseradish peroxidase-conjugated secondary antibodies (1:5000 dilution) at room temperature for one hour. The protein bands were visualized with Immobilon™ (Millipore). Actin was used as an internal control. The relative signal intensity was determined using ImageJ software.

### 2.3. Real-Time Quantitative Polymerase Chain Reaction (Real-Time PCR)

Trizol (Invitrogen, Carlsbad, CA, USA) reagent was used to isolate total RNA from the samples, according to the manufacturer’s protocol. The primers used for the detection of calsarcin-2 (Forward 5′-TGTTCAAGCTACGGCAGATG-3′, and Reverse 5′-GTAGGGCATTGCTGTCCTGT-3′) and Glyceraldehyde 3-phosphate dehydrogenase (GAPDH; Forward 5′-GCAAGAGAGAGGCCCTCAG-3′, and Reverse 5′-TGTGAGGGAGATGCTCAGTG-3′) were purchased from Genomics Technologies (New Taipei city, Taiwan). Data were analyzed by a StepOnePlus Real-Time PCR Detection System (Applied Biosystems, Foster City, CA, USA) using the SYBR Premix Ex Taq II kit (Takara Bio, Kyoto, Japan), according to the manufacturer’s instructions. GAPDH was used as an internal control for the quantification of relative mRNA expression levels with the 2^−ΔΔct^ method.

### 2.4. Cell Culture

The L6 myoblast cell line was purchased from the American Type Culture Collection and was verified by its short tandem repeat profile. The cells were maintained in Dulbecco’s modified Eagle medium (DMEM) (HyClone, Logan, UT, USA), supplemented with 10% fetal bovine serum (Hyclone) and 1% penicillin/streptomycin (Hyclone) at 37 °C in 5% CO_2_ in an air-humidified chamber. When the cells reached approximately 95% confluence, the growth medium was replaced with DMEM supplemented with 2% horse serum (Hyclone) and 1% penicillin/streptomycin to induce the differentiation of myotubes, according to the previous study [29].

#### 2.4.1. Lentiviral-Based Transfection and Delivery

The cells were plated on 24-well culture dishes at a density of 10^4^ cells/well. Lentiviral vectors encoding calsarcin-2 or short hairpin RNA targeted to calsarcin-2 (CS2^KD^) were purchased from OriGene (Rockville, MD, USA). The cultures were transduced with the lentiviral vectors to overexpress or knockdown calsarcin-2, as previously described [30].

#### 2.4.2. Identification of the Functional Annotation of Calsarcin-2 by Kyoto Encyclopedia of Genes and Genomes (KEGG) Analysis

The total RNA of the gastrocnemius muscle tissue collected from the chow and HFD groups of mice and L6 cells transfected with the lentiviral vector containing the *Rattus norvegicus* calsarcin-2 gene were harvested using a Trizol reagent. The array experiment was performed using the Agilent long non-coding RNA gene expression microarray technology (Agilent Technologies, Santa Clara, CA, USA), according to the manufacturer’s instructions. Briefly, the cDNA synthesized from total RNA was labeled by Cyanine-3-CTP using a Quick Amp Labeling Kit (Agilent Technologies), and the labeled cDNA was purified using an RNeasy Mini Kit (QIAGEN GmBH, Düsseldorf, Germany). The Agilent Gene Expression Hybridization Kit (Agilent Technologies) was then used for the hybridization. After adequate washing, the raw data of the microarray were obtained using an Agilent Microarray Scanner (Agilent Technologies). To determine the gene expression profile of calsarcin-2-overexpressing L6 cells, data from a long non-coding RNA gene expression microarray (Agilent Technologies) were used for a KEGG analysis. The top 10 categories (with gene counts and *p*-values) enriched in the KEGG analysis are presented.

#### 2.4.3. MTT Assay

After the transfection of the lentiviral vectors for 48 h, the cells (5 × 10^4^ cells per well) were seeded in a 96-well flat-bottom culture plate. One hundred μL of 0.2 mg/mL 3-(4,5-methylthiazol-2-yl)-2,5-diphenyl-tetrazolium bromide (MTT, USB Corporation, Cleveland, OH, USA) was added to each well, and the cells were incubated for three hours at 37 °C. After incubation, the MTT reagent was discarded, and 100 μL of dimethyl sulfoxide was added. The MTT was dissolved at room temperature for 20 min. Then, absorbance was measured with a Multiskan GO spectrophotometer (Thermo Scientific, Waltham, MA, USA) at a wavelength of 570 nm.

#### 2.4.4. Immunofluorescence Analysis

L6 myoblasts were plated in 6-well plates and fixed with 4% paraformaldehyde. The cells were then stained with anti-myosin heavy chain 2 (MYH) or calsarcin-2 antibodies (Abcam) at a dilution ratio of 1:100 at 4 °C overnight, followed by incubation with AlexaFluor-488- or AlexaFluor-594-conjugated secondary antibodies (Invitrogen) at a dilution ratio of 1:200 at room temperature for two hours. Negative controls included sections stained with a mouse universal negative control (Dako, Tokyo, Japan) and isotype control immunoglobulin G (Santa Cruz Biotechnology, Santa Cruz, CA, USA). Nuclei were visualized by staining them with 6-diamidino-2-phenylindole (DAPI) (Sigma-Aldrich). The samples were analyzed using fluorescence microscopy (Olympus, Hamburg, Germany).

### 2.5. Human Subjects

This study was approved by the institutional review board of National Cheng Kung University Hospital (A-ER-108-319), and all eligible participants signed an informed consent form before participating. From February 2019 to December 2021, a total of seventy-six human subjects in the endocrine/metabolism outpatient department were enrolled. The inclusion criteria consisted of adults aged 40 years and older. Those with the following diseases or conditions were excluded: (1) type 1 diabetes mellitus; (2) confirmed neuro-musculoskeletal disorders; (3) traumatic injuries or congenital anomalies of the upper or lower limbs; (4) current use of drugs that affect blood glucose levels, including glucocorticoids, thiazides, sympathomimetic drugs, and atypical antipsychotics; (5) acute coronary heart disease, cerebrovascular accident, or pancreatitis; (6) acute infection, such as pneumonia, urinary tract infection, soft tissue infection, or sepsis; (7) pregnancy; and (8) any other major diseases, including systemic inflammation or advanced malignant diseases.

Each subject’s body height and weight in light indoor clothing was measured. The body mass index (BMI) (in kg/m^2^) was calculated as the weight (in kilograms) divided by height squared (in meters squared). Muscle content was measured by a dual-energy X-ray absorptiometer (DXA; GE Lunar iDXA, GE Healthcare, Bucks, UK), and appendicular skeletal muscle mass index (ASMI) was determined as limb skeletal muscle mass divided by height squared (kg/m^2^). Hand grip strength was quantified by measuring the amount of static force that the hand can squeeze around a dynamometer (microFET^®^ HandGRIP dynamometers, Hoggan Scientific, Salt Lake City, UT, USA). In addition, we assessed gait speed by measuring 4 m gait speed at a normal pace. Moreover, all subjects underwent 12 h overnight fasting and blood sampling for a biochemical examination. Serum calsarcin-2 concentrations were measured using an enzyme-linked immunosorbent assay kit (intra-assay coefficient of variation (CV) < 10%, inter-assay CV < 12%) (MyBioSource). Plasma glucose concentrations were measured through the hexokinase method (Roche Diagnostic GmbH, Mannheim, Germany). Hemoglobin A1c (HbA1c) was determined via high-performance liquid chromatography (Tosoh Automated Glycohemoglobin Analyzer HLC-723 GHbV A1c 2.2, Tokyo, Japan, with an intra-assay CV of 0.5% and an inter-assay CV of 2.0%). Moreover, serum levels of total cholesterol, triglycerides, high-density lipoprotein cholesterol, and low-density lipoprotein cholesterol were determined in the central laboratory of National Cheng Kung University Medical Center with an autoanalyzer (Hitachi 747E, Tokyo, Japan). The estimated value of glomerular filtration rate (eGFR) (mL/min/1.73 m^2^) was calculated by the Modification of Diet in Renal Disease equation.

### 2.6. Statistical Analysis

The data were analyzed using the Windows version of the Statistical Package for the Social Sciences (SPSS version 21.0; SPSS, Chicago, IL, USA) [31]. For animal and cell experiments, Student’s two-tailed unpaired *t*-test or one-way analysis of variance (ANOVA) was used for comparison of the variables between each group, and the data were expressed as mean ± standard error (SEM). For the human study, continuous variables were expressed as means ± standard deviations (SD) and categorical variables as percentages. We conducted correlation tests, calculating Pearson’s correlation coefficient, to analyze the relationship between serum calsarcin-2 and muscle mass indexes in mice and humans. Additionally, a linear regression analysis was used to assess the association between serum calsarcin-2 concentration and human muscle mass. A *p*-value less than 0.05 was considered statistically significant.

## 3. Results

### 3.1. Establishment of Obese Sarcopenia Mouse Model

**Figure 1:**
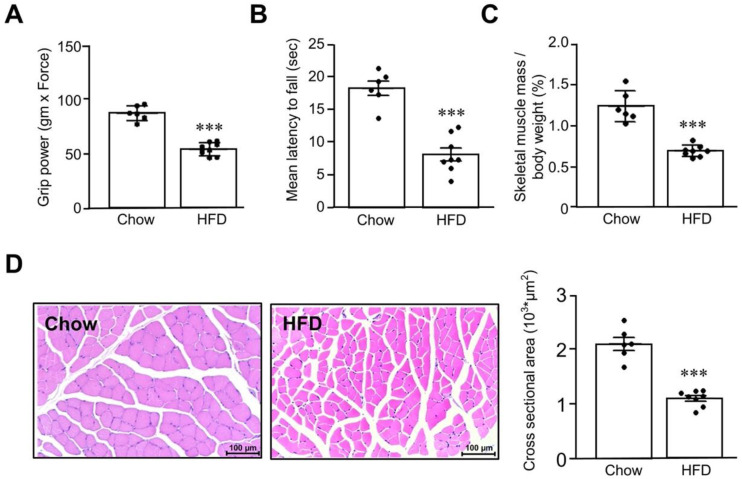
High-fat-diet-induced sarcopenia mouse model. Eight-week-old C57BL/6J male mice were fed a high-fat diet (HFD) or chow diet for 24 weeks. After a 12 h starvation period, grip strength (**A**) and latency to fall in the rotarod test were assessed (**B**). At the end of the experiments, the total mass of bilateral gastrocnemius muscle divided by body weight was measured (**C**). The cross-sectional area of gastrocnemius muscle fibers was determined using ImageJ (**D**). The black dots in the figures represent the assay values for each individual mouse. Quantification was performed on samples from 6 to 8 animals and is expressed as mean ± SEM. *** *p* < 0.001, as compared with chow diet group.

In order to determine the factors that contribute to the development of sarcopenia, we first established an HFD-induced sarcopenia mouse model, as described in a previous study [25]. After being fed with an HFD for 24 weeks, the body weight of the mice was significantly increased, as compared with the chow group (52.3 ± 8.1 g vs. 28.5 ± 1.4 g; *p* < 0.001). As shown in Figure 1, the mice fed with an HFD exhibited worse motor function, as determined by the grip strength (HFD: 56.3 ± 3.1 gm × Force, chow: 90.3 ± 4.9 gm × Force, *p* < 0.001) (Figure 1A) and rotarod tests (HFD: 8.1 ± 1.0 s latency to fall, chow: 18.3 ± 1.1 s latency to fall) (Figure 1B). Since gastrocnemius thickness is related to low skeletal muscle mass, we therefore investigated the changes of gastrocnemius muscle in the present study. The skeletal muscle mass index of the mice fed with an HFD was also significantly lower than that of chow group (HFD: 0.68 ± 0.02%, chow: 1.22 ± 0.08%, *p* < 0.001) (Figure 1C), while the cross-sectional area of the gastrocnemius muscle fibers was decreased (HFD: 1.02 ± 0.05 × 10^3^ μm^2^, chow: 2.08 ± 0.12 × 10^3^ μm^2^, *p* < 0.001) (Figure 1D).

### 3.2. Increased Calsarcin-2 Expression in HFD-Induced Sarcopenia Mouse Model

**Figure 2:**
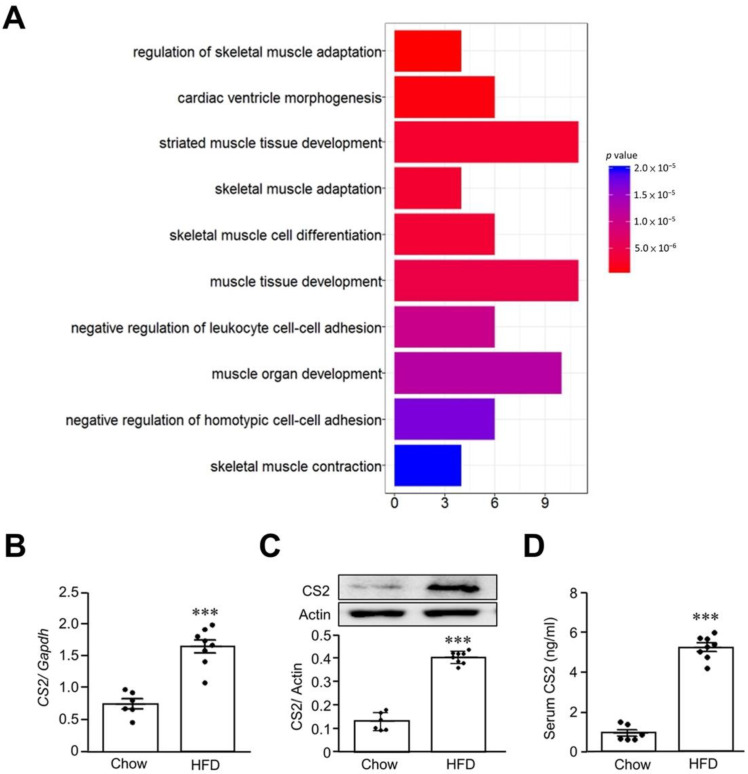
Calsarcin-2 is upregulated in sarcopenia mouse model. Eight-week-old C57BL/6J male mice were fed a high-fat diet (HFD) or chow diet for 24 weeks. After a 12 h starvation period, the skeletal muscle tissues were removed, the changes in gene profile were determined through a microarray, and the genes clusters were analyzed (**A**). The calsarcin-2 (CS2) mRNA expression via real-time PCR (**B**) and protein levels (**C**) via Western blots were determined. In addition, plasma samples were collected for measurements of CS2 concentrations by ELISA (**D**). The black dots in the figures represent the assay values for each individual mouse. Data are expressed as mean ± SEM. *** *p* < 0.001, as compared with chow diet group.

In order to clarify the possible factors that contribute to the development of sarcopenia, we used a microarray to analyze the changes in gene expression in skeletal muscle between the chow and HFD groups of mice. We found that approximately ~150 gene expressions were changed by twofold, and these genes were related to skeletal muscle differentiation, muscle tissue development, and skeletal muscle contraction (Figure 2A). Among these changed genes, calsarcin-2 was significantly increased, and we also confirmed the increased level of calsarcin-2 mRNA through real-time PCR (*p* < 0.001) (Figure 2B) and protein expressions by Western blots (*p* < 0.001) (Figure 2C) in the gastrocnemius muscle of the sarcopenia mouse model, as compared with the control group. In addition, the serum calsarcin-2 concentration in the sarcopenia mice was significantly increased in comparison to the chow group (*p* < 0.001) (Figure 2D), implying calsarcin-2 might play a role in sarcopenia.

### 3.3. Overexpression of Calsarcin-2 Promotes Differentiation of L6 Myoblasts

**Figure 3:**
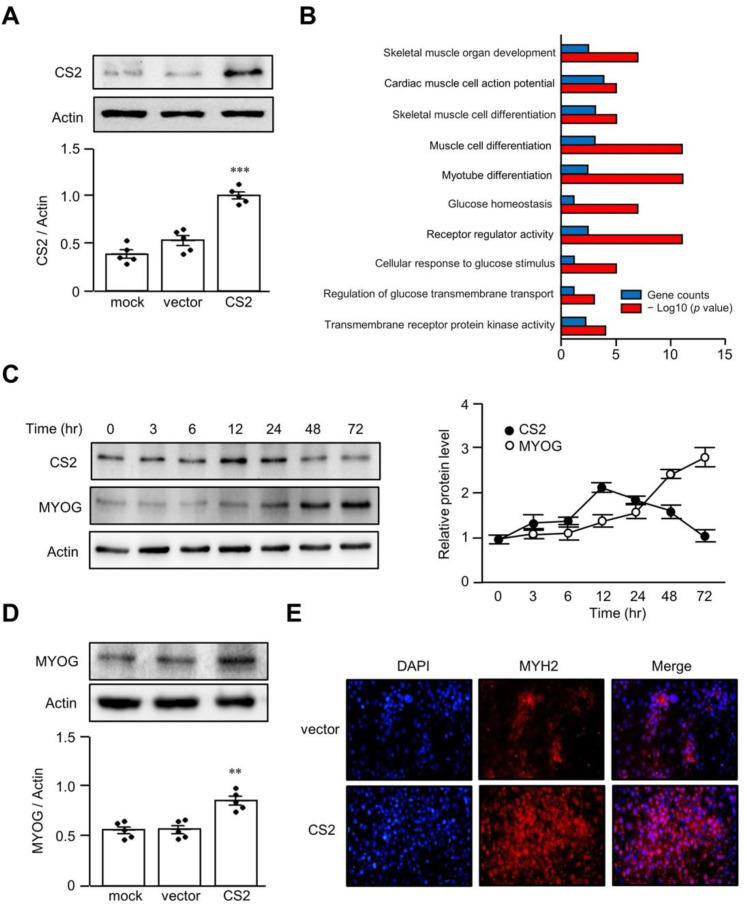
Overexpression of calsarcin-2 promotes myoblast differentiation. Overexpression of calsarcin-2 (CS2) in L6 myoblasts was accomplished using lentiviral vectors containing the CS2 gene (**A**). KEGG classification of differentially expressed genes in CS2-overexpressing L6 cells was performed (**B**). The differentiation of L6 myoblasts was induced, and protein lysates were harvested at indicated times for determination of CS2 and myogenin (MYOG) levels by Western blotting (**C**). The MYOG expression levels were determined in CS2-overexpressing L6 myoblasts (**D**). The expression of myosin heavy chain 2 (MYH) was determined in CS2-overexpressing L6 cells using immunohistochemistry (magnification 200×) (**E**). The black dots in the figures represent the assay values for each individual cell. Data are expressed as mean ± SEM. ** *p* < 0.01 and *** *p* < 0.001, as compared with indicated groups or control group.

To investigate the physiological role of calsarcin-2 in muscle, we established a calsarcin-2-overexpressing L6 cell model using a lentiviral vector (Figure 3A). The transcriptional profiles of the calsarcin-2-overexpressing L6 cells and controls were assessed via a microarray and compared through a KEGG pathway enrichment analysis. In the KEGG analysis, calsarcin-2 overexpression was associated with skeletal muscle organ development, skeletal muscle cell differentiation, myotube differentiation, glucose homeostasis, cellular response to glucose stimulus, and the regulation of glucose transmembrane transport (Figure 3B), implying that calsarcin-2 might participate in the differentiation of skeletal muscle. The expression of calsarcin-2 in L6 myoblasts during differentiation reached a peak at 12 h, while MYOG steadily increased up to 48 h, implying that calsarcin-2 might play a role as an initiator of myoblast differentiation (Figure 3C). In addition, we found that MYOG expression was elevated in the calsarcin-2-overexpressing L6 cells (Figure 3D), and myosin heavy chain 2 (MYH2) expression was elevated in calsarcin-2-overexpressing L6 cells after 48 h in the differentiation medium (Figure 3E).

### 3.4. Knockdown of Calsarcin-2 in L6 Cells Decelerated the Myoblast Differentiation

**Figure 4:**
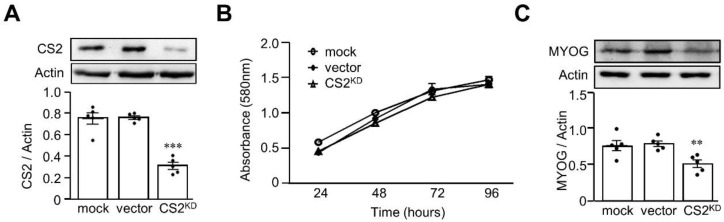
Knockdown of calsarcin-2 decelerated L6 myoblast differentiation. Knockdown of calsarcin-2 (CS2) in L6 myoblasts was accomplished using lentiviral vectors containing short hairpin RNA targeted to CS2 (**A**). Cell proliferation was assessed with the MTT assay (**B**). The MYOG expression levels were determined in CS2-knockdowned L6 myoblasts (**C**). Data are expressed as mean ± SEM. The black dots in the figures represent the assay values for each individual cell. ** *p* < 0.01 and *** *p* < 0.001, as compared with indicated groups or control group.

Not only is muscle strength important for the determination of sarcopenia, but muscle mass is also critical for its diagnosis. We then established a calsarcin-2 knockdown L6 cell model to confirm the effect of calsarcin-2 on myoblast proliferation (Figure 4A), and we found that the calsarcin-2 knockdown in L6 myoblasts had no significant effects on cell proliferation (Figure 4B). We then investigated the role of calsarcin-2 in myoblast differentiation, and we found that the expression of MYOG was significantly decreased in the L6 cells with calsarcin-2 knockdown (*p* < 0.01) (Figure 4C).

### 3.5. Serum Calsarcin-2 Concentration Was Negatively Associated with Skeletal Muscle Mass Index in Mice and Humans

**Figure 5:**
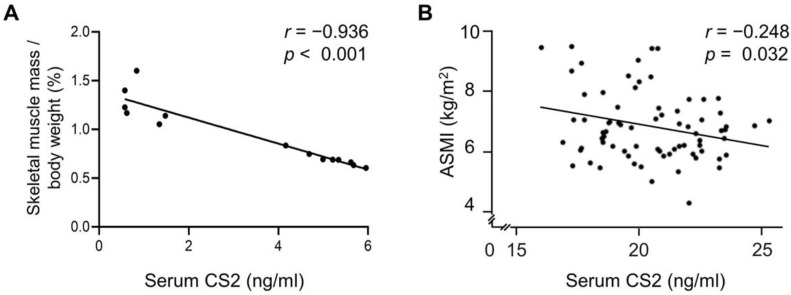
Serum calsarcin-2 concentration was negatively associated with skeletal muscle mass index in mice and humans. Eight-week-old C57BL/6J male mice were fed an HFD or chow diet for 24 weeks. The serum calsarcin-2 (CS2) concentrations were determined by enzyme-linked immunosorbent assay, and the total mass of bilateral gastrocnemius muscle mass was measured. The relationship between serum CS2 concentration and skeletal muscle mass per body weight (%) was analyzed using a linear regression analysis. The black dots in the figure represent the assay values for each individual mouse (**A**). A total of seventy-six human subjects were enrolled. The CS2 concentrations were determined by enzyme-linked immunosorbent assay, and the appendicular skeletal muscle mass index (ASMI) was determined by limb skeletal muscle mass (kg)/height^2^ (m^2^). The relationship between serum CS2 concentration and ASMI was analyzed using a linear regression analysis. The black dots in the figure represent the assay values for each individual human (**B**).

In order to clarify the relationship between serum calsarcin-2 and sarcopenia, the total mass of the bilateral gastrocnemius muscle of the mice fed a HFD or chow diet was measured. We found that serum calsarcin-2 concentration was negatively associated with the skeletal muscle mass per body weight (*r* = −0.936, *p* < 0.001) (Figure 5A). Similarly, a total of seventy-six human subjects were enrolled, and the clinical characteristics of the study subjects are shown in Table 1. We found that serum calsarcin-2 concentration was negatively associated with ASMI (*r* = −0.248, *p* = 0.032) (Figure 5). In addition, calsarcin-2 was independently associated with ASMI (β (95% CI) −0.537 (−1.840, −0.095), *p* = 0.03) after adjustments were made for age, gender, BMI, HbA1c, eGFR, and ALT (Table 2).

## 4. Discussion

To the best of our knowledge, this is the first study to investigate the role of calsarcin-2 in sarcopenia. We found that MYOG expression was increased in calsarcin-2-overexpressing L6 cells, whereas the knockdown of calsarcin-2 decreased the expression of MYOG, indicating that calsarcin-2 might play a role in skeletal muscle differentiation. Our findings were compatible with a previous study in which calsarcin-2 protein expression was found to have a positive regulatory effect on muscle growth and development in ducks [32].

The incidence of sarcopenia is swiftly escalating worldwide, paralleling the rises in both obesity and diabetes. A recent review succinctly outlined the robust interplay and detrimental cycles linking diabetes and sarcopenic obesity [33]. Insulin resistance promotes obesity; increasing adipocytes promotes cytokines to accelerate the catabolism of muscles; and loss of muscle reduces insulin-responsive tissues, which are complicated by insulin resistance [33]. Apart from promoting muscle cell differentiation, our cell model unveiled the involvement of calsarcin-2 in glucose metabolism, as indicated by the KEGG analysis. These encompass functions related to glucose homeostasis and cellular responses to glucose stimuli (Figure 3B). Furthermore, calsarcin-2 expression exhibited an increase in our HFD-induced sarcopenic mouse model, while the HFD mouse model was also utilized to simulate a type 2 diabetes model, given its association with insulin resistance and hyperglycemia [34,35]. Additionally, a prior human study similarly reported elevated calsarcin-2 expression among men diagnosed with metabolic syndrome [24]. Taken together, our findings suggest that calsarcin-2 may serve as a mediator connecting diabetes with the development of sarcopenic obesity. However, further investigation is warranted to delve deeper into this potential relationship.

In addition to the L6 myoblast model, an HFD-induced obese mouse model of sarcopenia was also used to investigate the role of calsarcin-2 in vivo. Intriguingly, increased calsarcin-2 expressions were seen in the sarcopenia mice, though calsarcin-2 positively regulated muscle cell differentiation in our cell model. Aging is a critical factor that contributes to the development of sarcopenia, but the animal model used in the present study was not an aging model. Therefore, human subjects with old age were enrolled, and the results were similar to the animal model in that ASMI was negatively associated with serum calsarcin-2 concentrations. While no prior study has delved into the physiological significance of serum calsarcin-2 concentration, our mouse study has illuminated a negative correlation between serum calsarcin-2 levels and an index of skeletal muscle mass (Figure 5A), as higher muscle calsarcin-2 expression was noted in HFD-induced sarcopenia mice (Figure 1C). This implies that serum calsarcin-2 might serve as a surrogate for assessing muscle calsarcin-2 protein levels, obviating the need for invasive muscle biopsies. We speculated that the increase in calsarcin-2 expression in vivo is a compensatory mechanism against sarcopenia, and thus increased serum calsarcin-2 concentrations were measured after muscle degradation.

Indeed, the human body employs various compensatory mechanisms to maintain equilibrium within specific ranges. As an illustration, in the event of heart failure, B-type natriuretic peptides undergo compensatory elevation, facilitating vasodilation and natriuresis as a response to fluid overload. This surge in B-type natriuretic peptides serves as a crucial diagnostic and prognostic indicator of heart failure [36]. In previous studies on sarcopenia, several compensatory factors have been identified. For instance, adiponectin is recognized for its role in safeguarding skeletal muscle against inflammation and injury, while sialic acid plays a crucial role in the maintenance of skeletal muscle integrity. The observed increase in the levels of adiponectin and sialic acid among sarcopenic patients is believed to act as a compensatory response to counteract muscle loss and the resulting decline in motor performance. As a result, both adiponectin and sialic acid have been incorporated as components of a comprehensive sarcopenic index [37]. Likewise, our in vivo and in vitro findings in this study indicate that the calsarcin-2 protein may assume a compensatory function in cases of sarcopenic obesity.

Previous studies on calsarcin-2 in humans have yielded disparate conclusions. Specifically, a downregulation of the calsarcin-2 protein was observed in healthy aging men, though this was not the case in men with sarcopenia [24]. Conversely, post-menopausal women exhibited an upregulation of the calsarcin-2 protein [38]. Our research endeavors to shed light on these contrasting findings. Given that menopause is known to expedite or precipitate sarcopenia [39], an upregulation of the muscle calsarcin-2 protein serves as a protective response against sarcopenia in this demographic. This compensatory effect, however, was not evident in healthy men. Consequently, our study suggests the potential diagnostic or prognostic significance of the muscle calsarcin-2 protein in sarcopenia. Additionally, serum calsarcin-2 levels may serve as a more accessible biomarker, owing to their robust positive correlation with the muscle calsarcin-2 protein.

## 5. Study Limitations

Our study has several limitations. Due to invasiveness, we were unable to obtain the muscle calsarcin-2 protein level in our human subjects. Consequently, we relied on the serum calsarcin-2 levels as a proxy. While there was a correlation between serum calsarcin-2 levels and muscle mass index, the precise metabolism of calsarcin-2 and the factors influencing its serum levels remain unclear. Furthermore, further evaluation is needed to confirm our current findings regarding the relationship between the muscle calsarcin-2 protein and sarcopenic obesity in humans.

## 6. Conclusions

In the present study, we found that calsarcin-2 is involved in skeletal muscle differentiation in a cell model. In addition, the increment in calsarcin-2 might have a compensatory effect on sarcopenic obesity, as determined by studies in mice and humans. However, the potential therapeutic applications of calsarcin-2 and details of its mechanisms still need further studies to be investigated.

## Figures and Tables

**Table 1:** Clinical characteristics of study participants.

N	76
Age (years)	61.8 ± 10.6
Numbers of male subjects	30
Number of subjects with diabetes	48
Body mass index (kg/m^2^)	25.34 ± 3.47
Waist circumference (cm)	89.8 ± 9.6
Fasting plasma glucose (mg/dL)	115.9 ± 26.4
Hemoglobin A1c (%)	6.50 ± 0.81
Serum calsarcin-2 (ng/mL)	20.4 ± 2.1
Estimated glomerular filtration rate (mL/min/1.73 m^2^)	84.4 ± 10.8
Alanine transaminase (U/L)	29.5 ± 15.4
Total cholesterol (mg/dL)	155.0 ± 33.7
High density lipoprotein–cholesterol (mg/dL)	53.2 ± 14.1
Low density lipoprotein–cholesterol (mg/dL)	96.0 ± 29.3
Triglyceride (mg/dL)	116.7 ± 56.6
Urine albumin to creatinine ratio (mg/g)	18.75 ± 21.10
Walking speed (m/s)	1.49 ± 0.38
Hand grip (gm × force)	23.59 ± 8.28
Appendicular skeletal muscle mass index (kg/m^2^)	6.84 ± 1.18

**Table 2:** Regression analysis between serum calsarcin-2 concentrations and clinical variables.

Variables	β (95% CI)	*p*
Age	−0.084 (−0.073, 0.039)	0.550
Gender	0.155 (−0.790, 2.132)	0.363
Body mass index	0.364 (−0.007, 0.454)	0.057
Hemoglobin A1c	0.086 (−0.407, 0.856)	0.481
Estimated glomerular filtration rate	0.083 (−0.031, 0.064)	0.495
Alanine transaminase	−0.205 (−0.063, 0.007)	0.109
Appendicular skeletal muscle mass index	−0.537 (−1.840, −0.095)	0.030

## Data Availability

The data that support the findings of this study are available from the corresponding author upon reasonable request.
